# Microglia in Alzheimer’s disease: from pathogenic mechanisms to therapeutic targeting

**DOI:** 10.3389/fimmu.2026.1952810

**Published:** 2026-10-26

**Authors:** Ling Tang, Xiao-Yan Li, Zhao Ran, Zhu-Zhu Qin, Shi-Yan Chen, Miao-Miao Zhang, Lin-Ying Zhou, Wen-Jun Wang, Liang Chen

**Affiliations:** 1 Department of Laboratory Medicine, Shifang People’s Hospital, Shifang, China; 2 Chongqing Key Laboratory of Sichuan-Chongqing Co-Construction for Diagnosis and Treatment of Infectious Diseases Integrated Traditional Chinese and Western Medicine, College of Medical Technology, Chengdu University of Traditional Chinese Medicine, Chengdu, China; 3 Department of Orthopedics, People’s Hospital of Deyang City, Deyang, Sichuan, China; 4 Department of Anesthesiology, Chengdu Shangjin Nanfu Hospital, Chengdu, Sichuan, China; 5 Yan’an Medical College, Yan’an University, Yan’an, Shaanxi, China

**Keywords:** Alzheimer’s disease, microglia, multiple sclerosis, neuroinflammation, pathogenic mechanisms, therapeutic targeting

## Abstract

Alzheimer’s disease (AD) is a multifactorial neurodegenerative disorder driven by β-amyloid (Aβ) deposition, tau hyperphosphorylation, synaptic dysfunction, neuroinflammation, vascular injury, and metabolic dysregulation. Recent advances in single-cell, spatial, and multi-omics transcriptomics have substantially reshaped our understanding of microglia, shifting the field from the classical M1/M2-like polarization dichotomy toward a model of state-dependent functional heterogeneity. Distinct subpopulations-including disease-associated microglia, lipid-associated microglia, interferon-responsive microglia, and senescence-associated microglia are now mechanistically linked to Aβ clearance, tau propagation, complement-mediated synaptic pruning, NOD-like receptor family pyrin domain containing 3 (NLRP3) inflammasome activation, and immunometabolic reprogramming. Central to this regulatory network are the TREM2-ApoE axis, complement cascades, NLRP3 inflammasome, mitochondrial dysfunction, and aberrant lipid/iron metabolism, which largely contribute to AD progression. In parallel, the clinical deployment of anti-Aβ monoclonal antibodies such as lecanemab has ushered in the era of disease-modifying therapy; however, limited efficacy and the occurrence of amyloid-related imaging abnormalities underscore that Aβ removal alone is insufficient to halt disease evolution completely. In this review, we systematically synthesize the evolving landscape of microglial states and their pathogenic contributions, and we further evaluate current therapeutic avenues, including microglia-targeted strategies, anti-Aβ antibody combination regimens, non-pharmacological interventions, and peripheral immunomodulation. We also compare these microglial mechanisms with those in multiple sclerosis to highlight shared neuroimmune modules and disease-specific features. Our goal is to provide a valuable updated reference for accurate molecular subtyping and multi-target combination therapy in AD.

## 1 Introduction

Alzheimer’s disease (AD) is an age-related neurodegenerative disorder characterized by progressive cognitive decline, memory loss, and behavioral impairment (1). As the leading cause of dementia, AD poses a major public health challenge amid global population aging. Global Burden of Disease 2019 estimates indicate that the global number of people with dementia will increase from 57.4 million in 2019 to 152.8 million by 2050, with the steepest increases in low and middle-income countries (2). Despite this immense burden, currently approved disease-modifying therapies (e.g., lecanemab, donanemab) are restricted to early-stage Alzheimer’s disease, and no treatment can completely halt or reverse full-blown AD progression, largely owing to its multifactorial and complex etiology (3, 4). Accumulating evidence has positioned microglia as central players in AD pathogenesis, and the disease is increasingly recognized as a neuroinflammatory condition intimately linked to aberrant microglial activation and neurodegeneration. Nevertheless, the precise mechanisms by which microglia contribute to AD remain incompletely understood.

Although multiple reviews have addressed microglia in AD, few systematically compare the full spectrum of newly-identified microglial states and their dynamic transitions. Pathogenic pathways are often described fragmentarily, while pharmacological and non-pharmacological interventions are usually discussed separately. To fill these gaps, this review: (i) characterizes diverse omics-derived AD-associated microglial subpopulations and clarifies their state interconversion; (ii) integrates disparate pathogenic signals into a coherent four-layer mechanistic network; (iii) synthesizes both pharmacological and non-pharmacological microglia-targeted strategies; (iv) highlights translational barriers including disease-stage dependency, APOE effects, and mouse-human inter-species divergence. This integrated perspective provides updated insights for mechanistic research and therapeutic development.

Microglial dysfunction is not unique to AD but rather represents a point of convergence for neuroinflammation across neurological disorders. Multiple sclerosis (MS), the prototypical neuroinflammatory disease of the central nervous system (CNS), engages many of the same microglial programs—including triggering receptor expressed on myeloid cells 2 (TREM2)-dependent activation, complement signaling, and inflammasome activity—albeit through a fundamentally different, autoimmune mode of initiation. We therefore directly compare the shared and divergent microglial mechanisms in AD and MS (Section 3.5 and **Table 1**), thereby situating this AD-centric synthesis within the broader neuroimmunology landscape.

**Table 1:** Comparative overview of microglial mechanisms in AD and MS.

Dimension	AD	MS	Relation
Primary driver	Aβ/tau proteinopathy with secondary innate immune activation	Autoimmune attack (autoreactive T/B cells) on myelin	Divergent
Typical onset/host context	Aging; immunosenescence (SAM prominent)	Young adults; relapsing–remitting or progressive	Divergent
DAM-like microglial states	DAM around Aβ plaques (TREM2-dependent)	DAM-like states in EAE and MS lesions	Shared
TREM2 axis	Drives homeostatic-to-DAM transition; Aβ containment	Promotes myelin debris clearance and remyelination	Shared (protective in both)
Complement/synaptic pruning	C1q–C3–CR3-mediated early synapse loss	C1q/C3-tagged synapse engulfment in demyelinated grey matter	Shared
NLRP3 inflammasome	Aβ- and tau-triggered amplification loop	Activated in lesions/EAE; blockade ameliorates disease	Shared
Lipid-laden microglia	LDAM: lipid droplets, impaired phagocytosis	Foamy microglia/macrophages in chronic active lesions	Shared
Iron/ferroptosis	Iron-loaded microglia at plaques; ferroptosis signatures	Iron-rimmed slowly expanding lesions	Shared
Type-I interferon/IRM	Chronic IFN-I signaling detrimental; IRM subset	IFN-β is an approved disease-modifying therapy	Divergent (opposite therapeutic valence)
Adaptive immunity crosstalk	Modulatory CD8+ T-cell subsets (GZMK+, CXCR6+PD-1+)	Disease-defining T/B cell autoimmunity; anti-CD20 effective	Divergent
Periphery/gut–brain axis	Infection, vascular injury, dysbiosis amplify pathology	Gut microbiota modulate EAE/MS activity	Shared

## 2 Microglial state lineages: from M1/M2 dichotomy to functional heterogeneity

### 2.1 The traditional M1/M2-like polarization model and its role in AD

Microglia are the resident immune cells and principal phagocytes of the CNS, playing critical roles in neural development, synaptic remodeling, tissue homeostasis, and immune surveillance (5–7). Under physiological conditions, they continuously survey the brain microenvironment and sustain homeostasis by clearing metabolic waste, engulfing cellular debris, and secreting neurotrophic factors (8). Upon exposure to infection, injury, or aberrant protein deposition, microglia undergo rapid morphological and functional remodeling—characterized by soma enlargement, process retraction, enhanced phagocytic activity, and robust release of inflammatory cytokines, chemokines, and other immune effectors (9, 10).

Historically, activated microglia have been classified into pro-inflammatory M1-like and anti-inflammatory M2-like states, thought to mediate tissue damage and repair, respectively. Accordingly, the classical M1/M2-like framework has long served as a simplified paradigm for interpreting the dual roles of microglia in neuroinflammation (11) (**Figure 1**). Throughout this review, we use M1-like and M2-like terminology only to describe this historical framework; otherwise, we refer to functional states such as pro-inflammatory, anti-inflammatory, and homeostatic-like states, as well as the disease-associated microglial subsets defined in Section 2.2. In AD, amyloid-β (Aβ) is recognized as a key initiator of microglial activation, driving proliferation, phagocytosis, and secretion of inflammatory mediators (12). During the early stages of the disease, microglia predominantly acquire an M2-like state, exerting neuroprotective effects through the clearance of Aβ deposits, inhibition of tau hyperphosphorylation, and production of neurotrophic and anti-inflammatory factors (13–15). However, as AD progresses, prolonged exposure to Aβ skews microglia toward an M1-like state, which releases copious pro-inflammatory cytokines (e.g., IL-1β, IL-6, TNF-α, and IFN-γ), thereby exacerbating neuroinflammation, synaptic damage, and neuronal loss (12, 16, 17). Concurrently, Aβ sustains microglial activation through engagement of membrane receptors including TREM2 (18), receptor for advanced glycation end products (RAGE) (19), and CD33 (20, 21), establishing a positive inflammatory feedback loop that accelerates AD pathology. In contrast, anti-inflammatory cytokines such as IL-4 and IL-10 promote microglial polarization toward an M2-like state via the STAT6/PPARγ signaling pathway, upregulating protective factors like TGF-β and IL-10, and thus enhancing tissue repair and neuroprotection (22).

**Figure 1:**
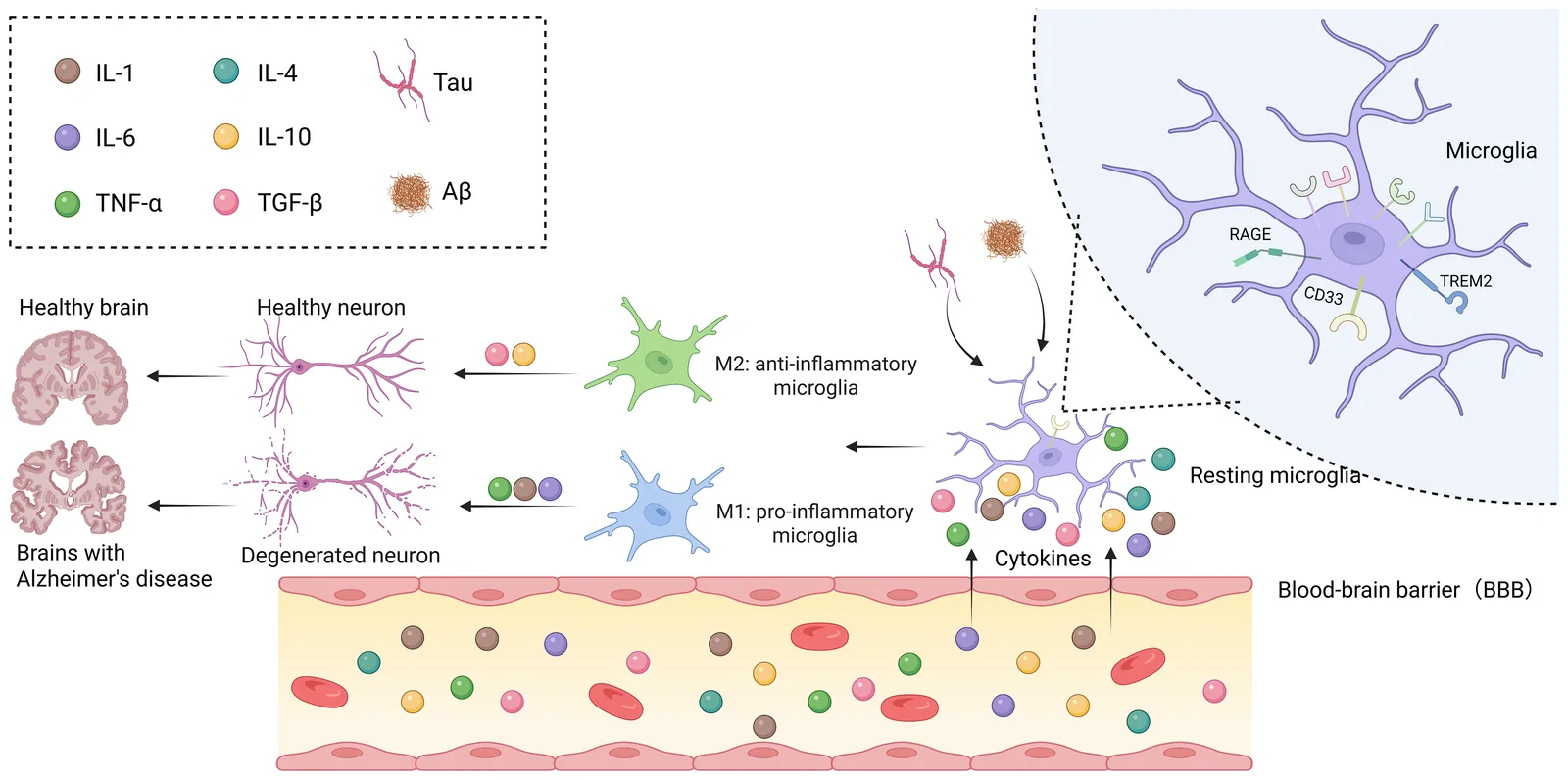
Historical M1/M2-like polarization model of microglia. Cytokines that have crossed the blood–brain barrier—including pro-inflammatory mediators such as IL-1, IL-6, and TNF-α and anti-inflammatory mediators such as IL-4, IL-10, and TGF-β—bind their respective cognate receptors on microglia. Microglial surface receptors such as RAGE, CD33, and TREM2 recognize distinct ligands and thereby modulate microglial activation and polarization. M1-like cells secrete pro-inflammatory mediators (IL-1, IL-6, TNF-α) that exacerbate neurodegeneration and promote AD pathology, whereas M2-like cells release neuroprotective factors (IL-10, TGF-β) to preserve neuronal integrity. Additionally, Aβ deposition and tau pathology further stimulate microglia, fueling a self-perpetuating inflammatory loop that accelerates the pathological cascade. This binary classification is an oversimplified historical framework and does not capture the full spectrum of microglial states in AD. Created with BioRender.com.

Substantial experimental evidence indicates that modulating microglial polarization can profoundly influence neuroinflammation and AD progression. For instance, loss of MER tyrosine kinase promotes pro-inflammatory polarization and exacerbates brain injury (23), whereas the mycobacterial adjuvant analogue trehalose-6,6’-dibehenate drives microglia toward an anti-inflammatory phenotype via PLC-γ1/PKC/ERK and AMPK signaling pathways, attenuating LPS-induced neuroinflammation (24). Naturally derived compounds have also shown regulatory potential: rosmarinic acid and quercetin suppress pro-inflammatory responses while upregulating M2-like markers (25–27); isovitexin promotes M2-like polarization through the CaMKKβ/AMPK-PGC-1α axis (28); and oxymatrine restores pro-inflammatory/anti-inflammatory balance and reduces inflammatory cytokines by inhibiting TLR4/NF-κB signaling (29). Collectively, these findings suggest that targeted regulation of microglial polarization confers meaningful neuroprotective benefits, offering novel avenues for immune-based therapeutic strategies in AD.

### 2.2 Limitations of the M1/M2 dichotomy and the paradigm shift in the single-cell era

Although the M1/M2 polarization model has provided a foundational framework for understanding microglia-mediated neuroinflammation, mounting evidence indicates that this binary classification inadequately captures the complexity of microglial dynamics *in vivo*. In neurodegenerative conditions such as AD, microglia do not adopt fixed polarization states; rather, they exhibit continuous, highly plastic functional phenotypes that vary across disease stages and brain regions in response to Aβ, tau, lipid dysmetabolism, and local microenvironmental cues (30). Indeed, numerous *in vivo* studies demonstrate that individual cells can simultaneously express both pro- and anti-inflammatory genes, with no clear demarcation between subsets—underscoring that the traditional dichotomy falls short of accurately reflecting AD microglial biology (**Figure 2**).

**Figure 2:**
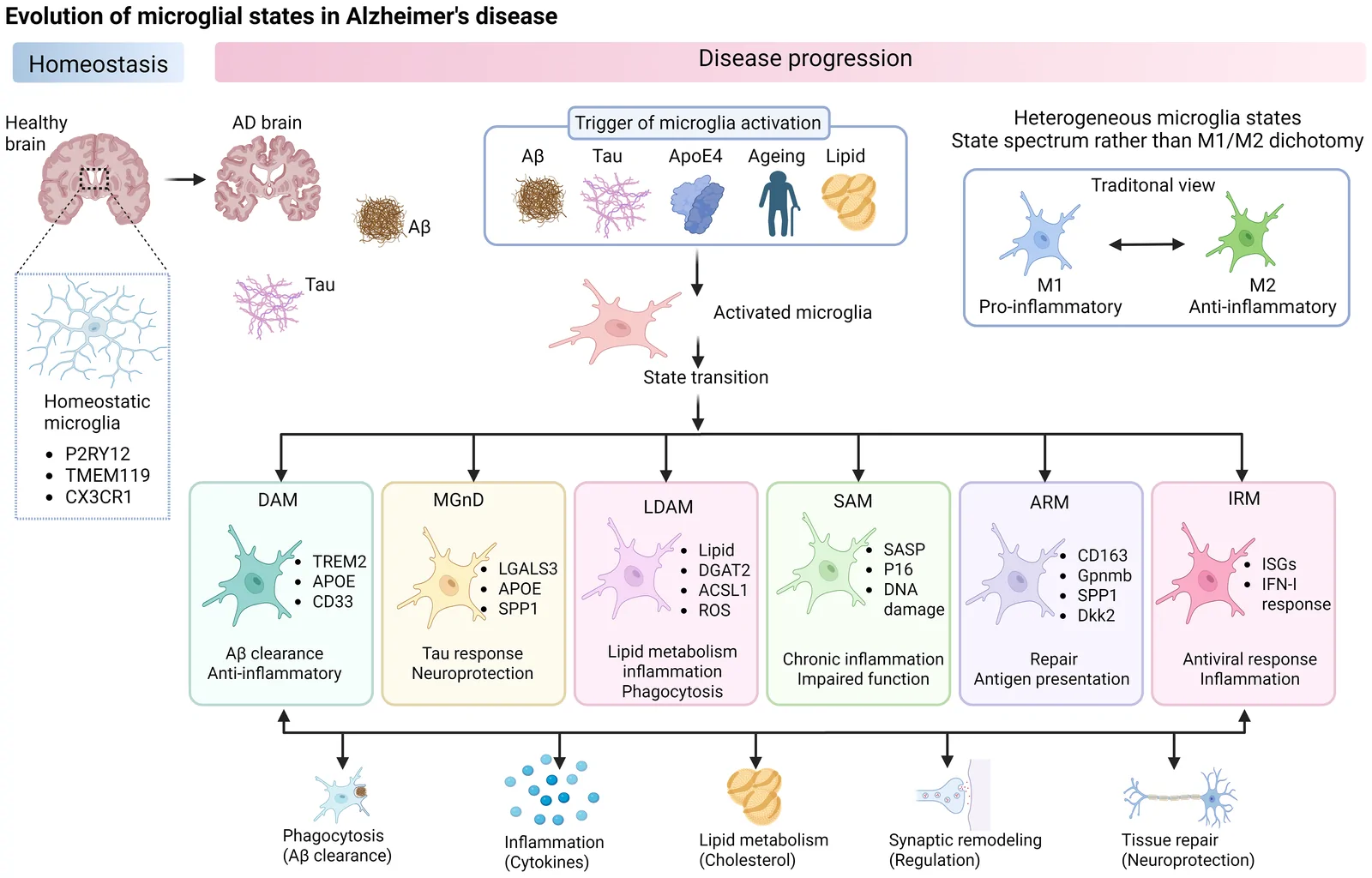
Heterogeneity and dynamic state transitions of microglia in AD. In the healthy brain, microglia perform homeostatic functions, including immune surveillance, metabolic waste clearance, and synaptic regulation. In AD, pathological drivers—including Aβ, tau, ApoE, aging, and lipid dysmetabolism—propel microglia away from homeostasis toward diverse disease-associated states. While the classical M1/M2-like framework broadly classifies these states as pro- or anti-inflammatory, single-cell and single-nucleus transcriptomic studies have revealed far greater heterogeneity, identifying subsets such as DAM, MGnD, LDAM, SAM, ARM, and IRM. These subpopulations participate in Aβ clearance, tau response, lipid metabolism, inflammatory regulation, and tissue repair, and they dynamically interconvert in response to disease stage and local microenvironmental cues, forming a continuous and highly plastic functional state lineage. Created with BioRender.com.

Recent advances in single-cell and single-nucleus RNA sequencing (scRNA-seq and snRNA-seq), together with spatial and multi-omics approaches, have provided unprecedented resolution for dissecting microglial heterogeneity in AD, as comprehensively summarized in recent systematic reviews (31, 32). Multiple studies have identified distinct transcriptomic subpopulations in both patients and mouse models (33–35), including disease-associated microglia (DAM) (18), the neurodegenerative phenotype (MGnD) (36), lipid-associated microglia (LDAM) (37), senescence-associated microglia (SAM) (38), and reactive subsets such as activated response microglia (ARM), transport-response microglia, and interferon-responsive microglia (IRM) (39). These subsets are not static; instead, they participate in—and transition across—key processes including pathological clearance, lipid regulation, inflammatory amplification, repair, and immune senescence, collectively forming a dynamic continuum shaped by genetic, metabolic, and microenvironmental inputs. Consequently, this functional heterogeneity–centered paradigm more faithfully represents the authentic characteristics of AD microglia than the conventional M1/M2-like classification, and offers a refined framework for stage-specific therapeutic targeting. Accordingly, the following sections focus on the principal microglial subpopulations and their distinct roles in AD pathogenesis.

### 2.3 Major functional subsets of AD-associated microglia

DAMs are the best-studied AD-related microglial subset enriched around Aβ plaques in mouse models and human AD brains (18, 40). They express high AD-risk genes including *TREM2*, *APOE*, and *CD33* (41). DAMs mediate Aβ phagocytosis and confer neuroprotection at early disease stages; as pathology progresses, phagocytic function declines while pro-inflammatory programs are sustained, leading to context-dependent protective or detrimental outcomes (40). While DAMs are primarily triggered by Aβ pathology, other microglial subsets emerge in response to tau-driven neurodegeneration. MGnD represents a tau-responsive microglial subset (36). They can clear phosphorylated tau and complement-tagged synapses to restrict tau spread, yet activated MGnD may release tau-containing extracellular vesicles that propagate tau pathology (36, 42). Their final functional output depends on disease stage and local microenvironment.

Unlike DAM and MGnD, which primarily mediate pathological protein clearance, LDAMs represent a metabolically reprogrammed state in chronic neuroinflammation, defined by abundant intracellular lipid-droplet accumulation (37). This subset exhibits disrupted lipid homeostasis, mitochondrial defects, and impaired phagocytosis. LDAMs are detected in aged and AD brains, and their formation correlates with APOE status, cholesterol dyshomeostasis, oxidative stress, and persistent inflammation (43).

Aging is not only a major risk factor for AD onset and progression but also a key regulatory dimension driving microglial state transitions. SAMs are characterized by elevated cellular senescence markers and impaired immune function, driven by chronic inflammation, DNA damage, and metabolic disturbance (38). Microglial senescence promotes DAM formation and Aβ pathology, and AD-risk alleles aggravate senescence-related neurodegeneration in tauopathy models (44). SAMs secrete senescence-associated pro-inflammatory mediators, sustaining neuroinflammation and reducing phagocytic and tissue-repair capacity.

Beyond the subsets described above, reactive microglia represent a dynamic functional state further subdivided by single-cell transcriptomic studies, with ARM and IRM being the most prominent. ARMs respond to Aβ plaques and express MHC-II and tissue-repair-associated genes; APOE4 or TREM2 risk variants reduce protective CD163^+^ ARM populations in AD patients (39). IRMs display prominent interferon-stimulated-gene signatures modulated by factors including miR-34a (45). Additional subsets such as vascular-associated microglia and homeostatic-responsive hippocampal microglia have also been reported in AD-relevant datasets (46, 47). Collectively, these findings underscore that the microglial state network in AD is far more complex than currently appreciated, necessitating further single-cell and spatial transcriptomic investigations to decipher its spatiotemporal dynamics. **Table 2** summarizes the defining features, principal triggers, functions, and disease relevance of these major AD-associated microglial subsets.

**Table 2:** Defining features of the major AD-associated microglial subsets.

Subset	Key defining markers/features	Principal triggers	Principal functions	Stage- and disease-relevance
DAM	TREM2/APOE-dependent program (APOE, Cst7, Lpl, Itgax); enriched AD risk genes (TREM2, APOE, CD33)	Aβ plaques; tau pathology; neurodegenerative cues	Plaque encapsulation and Aβ phagocytosis; modulation of local inflammation	Protective early, wanes as disease advances; most characterized subset
MGnD	Neurodegeneration-induced program overlapping DAM (APOE, Lpl, Clec7a); TREM2-dependent	Tau aggregates (NP-tau); complement-opsonized synapses	Recognition and uptake of p-tau; release of p-tau-bearing extracellular vesicles	Dual-edged: tau clearance versus tau propagation
LDAM	Lipid droplet accumulation; ACSL1; DGAT2; impaired phagocytosis	Aging; APOE4; cholesterol dyshomeostasis; high-fat diet; oxidative stress	Lipid storage; ROS production; pro-inflammatory signaling	Links immunometabolic failure to chronic neuroinflammation
SAM	Senescence markers; SASP (IL-1β, IL-6, TNF-α, chemokines); DNA damage; cGAS activation	Replicative senescence; chronic inflammatory stimulation	SASP-driven inflammatory amplification; weakened phagocytosis and tissue repair	Promotes DAM emergence and Aβ pathology in the aging brain
ARM	MHC-II; repair-associated genes (Dkk2, Gpnmb, Spp1); AD risk genes	Aβ plaque deposition	Pathological recognition; tissue repair programs	CD163^+^ ARM responses are attenuated by APOE4 and TREM2 risk variants
IRM	Interferon-stimulated gene expression	Type I interferon signaling; miR-34a deficiency	Inflammatory activation; nucleic acid sensing	miR-34a loss improves cognition and lowers the Aβ42/Aβ40 ratio in models

In conclusion, microglia in the AD brain do not adopt a single activated state but instead constitute a highly heterogeneous functional network encompassing DAM, MGnD, LDAM, SAM, and diverse reactive subsets. These subsets execute distinct biological processes and exert stage-dependent roles throughout AD progression. Moving past the oversimplified M1/M2 dichotomy towards state-dependent heterogeneity reshapes our understanding of microglia biology in AD. The molecular drivers governing these diverse phenotypic shifts constitute a core component of AD pathogenic mechanisms.

## 3 Core microglial mechanisms in AD pathogenesis: a multi-level network and cross-disease integration with MS

Microglial dysfunction has emerged as another critical pillar of AD pathogenesis. Rather than operating through a single linear cascade, recent evidence reveals that microglia engage in a complex, multi-layered mechanistic network encompassing four interrelated levels of functional progression: First, at the pathological recognition and subset specification level (Section 3.1), microglia sense Aβ and tau stimuli via the TREM2–ApoE axis, initiating the transition toward DAM. This initial response dictates their subsequent functional trajectory. Second, at the effector level (Section 3.2), activated microglia drive excessive complement-mediated synaptic pruning and amplify inflammatory cascades through the NOD-like receptor family pyrin domain containing 3 (NLRP3) inflammasome, directly contributing to synaptic injury and pathological tau dissemination. Third, as inflammatory signals accumulate, microglia enter a state of homeostatic collapse (Section 3.3), characterized by metabolic reprogramming, mitochondrial dysfunction, aberrant lipid droplet accumulation, and ferroptosis—culminating in impaired phagocytic capacity and functional disintegration. Finally, these intracerebral processes are exacerbated by a microenvironmental amplification layer (Section 3.4), wherein peripheral insults—including infection, vascular injury, and gut microbiota dysbiosis—reshape the central immune milieu via the brain–gut axis and immune cell infiltration, establishing a synergistic vicious cycle. Collectively, these four levels constitute a dynamic progression chain—from initial perception to effector execution, homeostatic collapse, and peripheral amplification—interlinked by positive feedback and cross-talk that collectively construct the core mechanistic framework by which microglia contribute to AD progression (**Figure 3**). To determine which of these mechanisms constitute a conserved neuroimmune core and which are disease-specific, the fifth level (Section 3.5) distinguishes them by comparing microglial mechanisms in AD and MS (**Table 1**).

**Figure 3:**
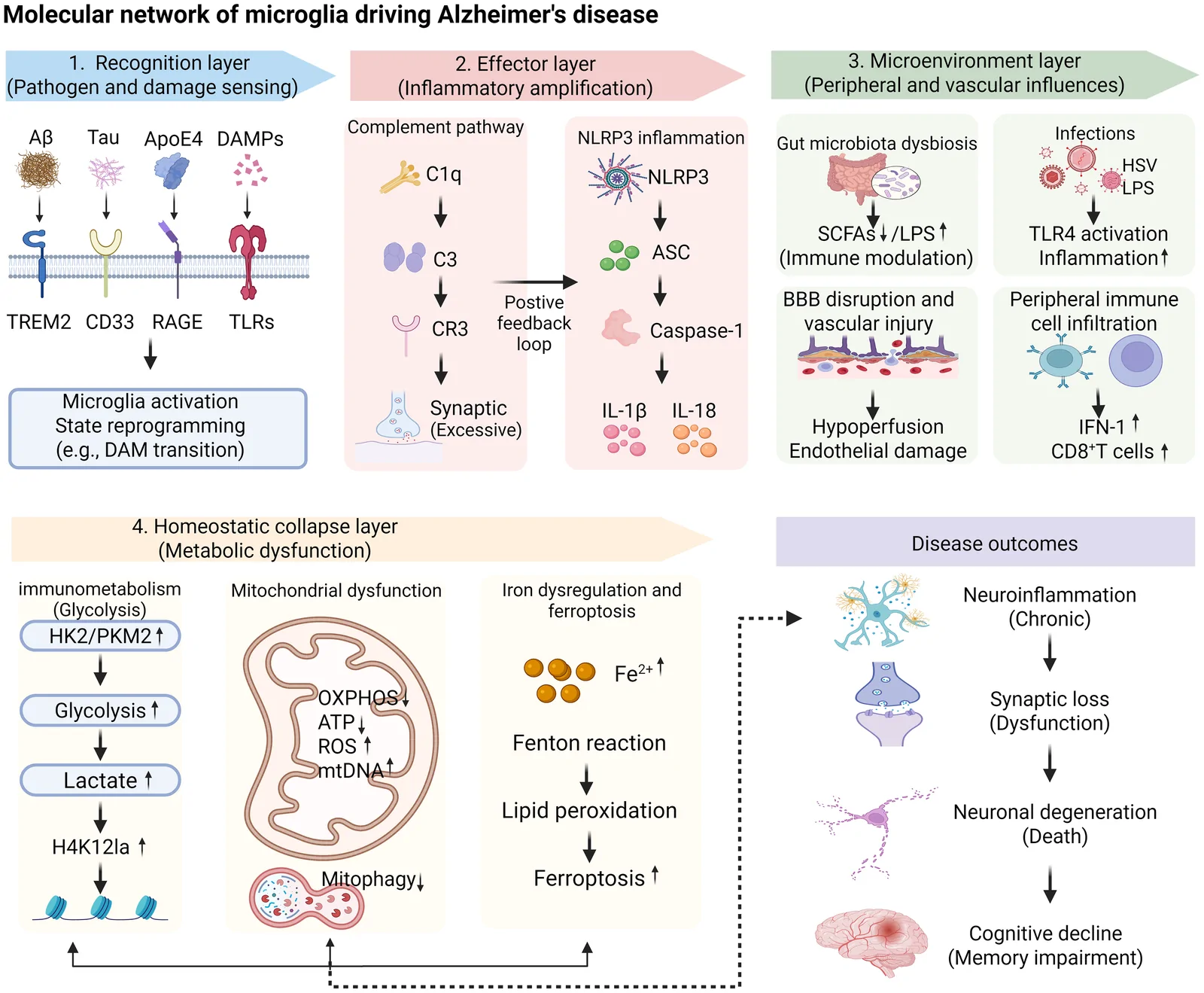
Multi-level mechanistic network of microglial involvement in AD pathogenesis. Aβ, tau, genetic, and environmental factors drive microglial state remodeling across four interrelated levels: (I) pathological perception, (II) effector execution, (III) homeostatic collapse, and (IV) microenvironmental amplification. At the perception level, Aβ, tau, ApoE, and DAMPs engage TREM2, CD33, RAGE, and TLRs, with the TREM2–ApoE axis regulating DAM transition. At the effector level, complement C1q–C3–CR3 mediates synaptic pruning, while NLRP3–ASC–caspase-1 inflammasome drives IL-1β/IL-18 release to amplify neuroinflammation. At the homeostatic collapse level, immunometabolic reprogramming, mitochondrial dysfunction, lipid droplet accumulation, and ferroptosis interact to progressively impair microglial phagocytosis and metabolism. At the amplification level, peripheral infection, vascular injury, gut dysbiosis, and immune infiltration remodel the brain microenvironment and intensify inflammation. These interconnected mechanisms form a self-reinforcing feedback loop that ultimately promotes chronic neuroinflammation, synaptic loss, neurodegeneration, and cognitive decline. Created with BioRender.com.

### 3.1 Pathological perception and subset specification: the ApoE–TREM2 axis and Aβ–tau synergistic responses

The amyloid cascade hypothesis posits that Aβ aggregation serves as a core initiating event in AD pathogenesis (48). As damage-associated molecular patterns (DAMPs), Aβ oligomers and fibrils trigger innate immune activation in microglia. Sustained Aβ exposure disrupts immune homeostasis: while microglia-derived apoptosis-associated speck-like protein containing a CARD (ASC) specks seed Aβ fibrillization to fuel inflammatory-amyloid feedback, microglia are recruited to Aβ deposits and actively phagocytose amyloid at the early stage of plaque formation (49). However, this prolonged Aβ challenge skews microglia toward a pro-inflammatory phenotype and drives persistent neuroinflammation (49, 50). Beyond Aβ deposition, pathological tau remodels the microglial transcriptional landscape. Microglial exosomes represent key vectors mediating tau spread; inhibition of microglia or exosome biogenesis restrains tau propagation (51). The NLRP3 inflammasome bridges Aβ-related inflammation and tau hyperphosphorylation (52, 53). Adequate DAM transition restricts Aβ-driven tau dissemination, highlighting the importance of microglial responses for limiting tau pathology (54, 55).During pathological sensing, the TREM2-ApoE axis determines microglial subset fate. TREM2, a microglia-specific immune receptor and one of the most significant AD risk genes, plays a critical role in maintaining homeostasis, facilitating Aβ clearance, and regulating immunometabolism (56). DAM maturation proceeds in two phases: TREM2-independent down-regulation of homeostatic genes, followed by TREM2-driven activation of phagocytic, lipid-metabolic, and inflammatory programs (18). However, TREM2 is not a purely protective factor. Although the TREM2 T96K variant has been reported to enhance ligand-dependent activation in some assays, its net effect is context-dependent, and it is not simply a gain-of-function mutation; it paradoxically reduces plaque-associated microglial aggregation, lowers soluble TREM2 levels, impairs Aβ uptake, hinders the homeostatic-to-DAM transition in 5×FAD mice, and interferes with IL-6/JAK/STAT3, complement, and IFN-γ signaling pathways (57).

Conversely, ApoE—the primary brain lipid carrier—exists in an E4 isoform identified as a driver of multifaceted AD damage, conferring both neurotoxic properties and a loss of neuroprotective functions (58). The “APOE cascade hypothesis” further underscores its centrality in age-related cognitive decline and AD progression, impacting not only Aβ deposition but also indirectly modulating tau pathology via lipid metabolism and immune response regulation (59, 60). Selective astrocytic APOE4 ablation mitigates tau-driven neurodegeneration and aberrant synaptic pruning by microglia (61). Recent work shows APOE4 suppresses protective MGnD signatures via the ITGB8–TGFβ–inositol polyphosphate-5-phosphatase D checkpoint and weakens microglia-astrocyte crosstalk, reducing Aβ encapsulation and clearance efficiency (62).

Beyond Aβ, tau, and the TREM2–ApoE axis, sex and APOE genotype act as additional modifiers of microglial state transitions in AD. Sex differences are increasingly recognized in microglial responses: female AD patients and APP/PS1 mice show enrichment of IFN-response genes, with microglia as the primary effector cells, and enhanced IFN-I signaling promotes neuroinflammation and neurodegeneration (63). Microglial metabolism also displays sexual dimorphism relevant to AD pathology (64). These sex effects intersect with APOE genotype: APOE4-related risk is more pronounced in women, and sex-dependent APOE4 neutrophil–microglia interactions drive cognitive impairment (65), while AD-linked risk alleles elevate microglial cGAS-associated senescence and neurodegeneration in tauopathy models (44). Therefore, sex and APOE genotype should be jointly considered in patient stratification and microglia-targeted therapeutic design. Recent reviews have systematically addressed the intersection of sex and APOE genotype in microglial biology, highlighting how female sex and APOE4 jointly shape microglial inflammatory responses and disease progression (66). Taken together, microglial functional outputs are shaped jointly by pathological stimuli and intrinsic genetic risk background.

### 3.2 Effector layer: complement-mediated synaptic pruning and NLRP3 inflammasome amplification

Upon sensing Aβ and tau, microglia propagate neuroinflammatory cascades through two core effector systems: the complement cascade and the NLRP3 inflammasome. These pathways not only mediate synaptic damage, inflammatory cytokine release, and impaired clearance of pathological proteins but also engage in mutual reinforcement, forming a persistent positive feedback loop that amplifies AD pathology (67). Critically, microglial effects extend beyond autonomous actions. Microglia-derived IL-1α, TNF, and C1q drive astrocytes toward a neurotoxic A1 phenotype, depriving them of their supportive functions for synapses and neurons while actively releasing factors that kill neurons and oligodendrocytes (68).

Early in AD, aberrant complement activation leads to C1q deposition at synapses, where it acts as a phagocytic ‘eat-me’ signal. Engagement of the microglial receptor CR3 then drives excessive synaptic pruning, directly precipitating early cognitive decline (69, 70). This complement activity is context-dependent: beneficial for debris clearance under homeostasis, but neurotoxic upon chronic over-activation. The δ-opioid receptor (DOR) counter-regulates C1q-driven complement over-activation and synaptic loss (70). Systemic insults such as *Porphyromonas gingivalis* infection amplify C1q-mediated microglial injury (71, 72). Notably, the complement cleavage product C3a engages its receptor C3aR to trigger mitochondrial dysfunction and a reactive oxygen species (ROS) burst in microglia, promoting synaptic loss and cognitive decline in an NLRP3-dependent manner (73). This C3a–C3aR–NLRP3 axis reveals that many complement-mediated pathological effects are amplified through downstream NLRP3 signaling, tightly coupling the two mechanisms.

The NLRP3 inflammasome serves as the core actuator and signal convergence point for the inflammatory cascade. As classic DAMPs, Aβ fibrils directly induce NLRP3 assembly, activating caspase-1 and driving IL-1β and IL-18 release (74). More importantly, NLRP3 acts as a molecular bridge between Aβ and tau pathologies: tau triggers Galectin-9 to engage TLR4, activating NLRP3 and promoting inflammatory factor release and tau spread (75). Complement and NLRP3 form a feed-forward amplification loop. Therefore, interrupting their mutual co-amplification may be more therapeutically promising than targeting either pathway alone.

### 3.3 Homeostasis breakdown layer: immune metabolic reprogramming, mitochondrial dysfunction, and ferroptosis

Metabolic reprogramming is not merely a secondary by-product of microglial activation but a core driver of phenotypic shift in AD. Plaque-associated microglia shift toward enhanced glycolysis; accumulated lactate induces histone H4K12 lactylation, further up-regulating PKM2 and sustaining pro-inflammatory activation (76). Similarly, TBG096, a natural compound with NGF-mimetic activity, simultaneously inhibits glycolytic rate-limiting enzymes, activates AMPK, and enhances chaperone-mediated autophagy via the Hsc70/HK2/PKM2/LAMP2A axis. This alleviates glial activation, attenuates Aβ and tau pathology, and promotes neurogenesis, revealing an intrinsic link between metabolic regulation, autophagic repair, and neuronal regeneration (77). However, this metabolic shift toward glycolysis is not an isolated phenomenon; rather, it inextricably involves mitochondrial metabolism, as heightened glycolytic flux typically comes at the cost of oxidative phosphorylation (OXPHOS) and drives deeper mitochondrial impairment.

Mitochondrial homeostasis damage represents a critical node in overall systemic breakdown. Physiologically, microglia rely on OXPHOS to fuel migration, phagocytosis, and immune surveillance. In the AD brain, however, sustained inflammation and pathology drive the aforementioned glycolytic shift, which suppresses OXPHOS and triggers mitochondrial damage, ATP depletion, and ROS accumulation. These factors form a vicious cycle that severely compromises Aβ clearance and perpetuates homeostatic failure. Indeed, damaged mitochondria accumulate in AD microglia, and preserving mitochondrial mass enhances phagocytic efficiency (78). Ulland et al. demonstrated that TREM2 deficiency leads to excessive mTOR inhibition and metabolic defects, whereas creatine analogue (cyclocreatine) supplementation or mTOR activation partially restores OXPHOS and Aβ plaque clearance (79). Baik et al. further established that persistent mTOR inactivation underlies reduced phagocytosis in AD microglia, and restoring mTOR activity significantly improves Aβ clearance (80). Similarly, IL-33 promotes mitochondrial activity and microglial phagocytosis via AKT-dependent mechanisms, reinforcing mitochondrial fitness as a core determinant of microglial function (81). Critically, mitochondrial dysfunction not only curtails energy supply but also generates ROS, which directly perturbs lipid metabolism and induces pathological lipid droplet accumulation.

Glycolytic reprogramming and mitochondrial injury jointly promote pathological changes in lipid metabolism. Up-regulated DGAT2 drives triglyceride synthesis and lipid-droplet biogenesis, impairing Aβ phagocytosis (82). ACSL1 defines a distinct microglial state closely linked to lipid-droplet accumulation (83). Concurrently, the TRPV1–PKM2–SREBP1 signaling axis serves as a critical rheostat for microglial lipid homeostasis; activating TRPV1 suppresses PKM2 dimerization and SREBP1 activation, significantly reversing the LDAM phenotype in 3×Tg mice (84). Collectively, these findings delineate a coherent pathogenic cascade from initial metabolic reprogramming to lipid droplet accumulation and, ultimately, to phagocytic failure.

Metabolic and mitochondrial disturbances are compounded by metal ion homeostasis imbalance. Within AD plaques, accumulated iron, copper, and zinc drive Aβ aggregation and ROS production, intensifying oxidative stress and neuronal injury (85). Iron deposition, specifically localized to microglia surrounding plaques, correlates strongly with tau pathology (86, 87). Copper, via the Cu (II)–Aβ complex, exacerbates microglial activation, drives TNF-α and NO production to amplify neurotoxicity (88), while simultaneously suppressing phagocytosis and skewing cells toward a pro-inflammatory phenotype (89, 90). Transcriptomic analyses reveal robust ferroptosis signatures in AD brains, mediated in part by activation of the integrated stress response, eIF2α phosphorylation, and ATF4 signaling, which collectively intensify cellular stress and neurodegeneration (91). Autophagic dysfunction further fuels microglial ferroptosis: microglia-specific *Atg7* deletion impairs the unfolded protein response, heightens oxidative stress and lipid peroxidation, induces ferroptosis, and compromises Aβ plaque containment, thereby exacerbating neurotoxicity (92). In contrast, ghrelin can up-regulate ferroptosis-suppressing molecules via BMP6/SMAD1 signaling, mitigate lipid peroxidation, and restore more protective microglial phenotypes (93). Taken together, glycolytic shift, mitochondrial impairment, pathological lipid droplet accumulation, and iron-dependent ferroptosis form a self-reinforcing downward cascade that pushes microglia towards homeostatic collapse.

### 3.4 Microenvironment amplification layer: peripheral inflammation, vascular injury, and gut–brain–immune crosstalk

Beyond intrinsic CNS pathology and microglial immunometabolic dysregulation, extrinsic microenvironmental factors—notably infection and vascular injury—profoundly modulate microglial function by reshaping the central immune landscape. The microbial/infection hypothesis posits that pathogen-driven chronic inflammation promotes AD pathology. Herpes simplex virus type 1 transiently phosphorylates Tau and increases high-molecular-weight Aβ oligomer accumulation (94, 95). On the vascular front, chronic cerebral hypoperfusion damages endothelial cells, disrupts neurovascular unit homeostasis, and triggers aberrant microglial activation (96, 97); post-ischemic endothelial necroptosis mediates vascular injury, while activated microglia further amplify neuronal apoptosis and inflammatory responses (98). These systemic-level drivers do not act in isolation. Rather, central pathological signals are tightly interwoven with the peripheral immunometabolic network. The microbiota–gut–brain–immune crosstalk serves as a key signaling conduit linking these compartments.

Gut microbiota influence neurodegenerative progression by modulating immune metabolism, inflammatory responses, and microglial function. In APP/PS1 mice, microbial-derived short-chain fatty acids alter microglial activation states and affect Aβ levels, implicating gut metabolites as key intermediaries between the peripheral environment and central immune responses (99). Conversely, gut dysbiosis compromises intestinal barrier integrity, promotes peripheral inflammatory factor release, and—via the microbiota–gut–brain–immune crosstalk—amplifies neuroinflammation, accelerating AD pathology (100, 101). These findings suggest that dietary modulation or targeted regulation of intestinal microecology may offer novel preventive and therapeutic strategies. Crucially, however, gut-derived signals must be relayed through immune cells to reshape the central microenvironment. The direct interaction between the peripheral immune system and brain-resident microglia thus constitutes the final execution step, bridging peripheral cues with central pathological responses.

At the immune-execution level, peripheral inflammatory signals are relayed to the CNS through both innate and adaptive immune pathways, thereby amplifying microglial dysfunction. Microglial type I interferon (IFN-I) signaling serves as a key driver of AD neuroinflammation, with notable sexual dimorphism. Transcriptomic analyses reveal significant enrichment of IFN-response genes in female AD patients and APP/PS1 mice, with microglia identified as the primary effector cells. Enhanced IFN-I signaling promotes neuroinflammation and neurodegeneration, whereas cGAS–STING pathway blockade attenuates these effects and improves cognition, positioning the cGAS–STING/IFN-I axis as a critical link between cellular injury signals and microglial inflammatory responses (63). On the adaptive immunity front, microglia initially restrict phosphorylated Tau (pTau) spread in early pathology. However, as disease progresses, they transition into functionally impaired antigen-presenting cells that recruit granzyme K (GZMK)-expressing CD8^+^ T cells. This subset, regulated by immune checkpoints such as PD-1 and TIGIT, limits pathological microglial accumulation and inhibits Tau dissemination (102). Further studies identify CXCR6^+^PD-1^+^CD8^+^ T cells in AD brains and model mice, closely co-localized with plaque-associated microglia and interacting via CXCL16–CXCR6 signaling. Depletion of CXCR6 or CD8^+^ T cells reduces brain-resident CD8^+^ T cell accumulation, triggers microglial pro-inflammatory factor release, and exacerbates Aβ deposition and cognitive decline, implicating resident T cells in AD immune homeostasis (103). Nevertheless, CD8^+^ T cells are functionally heterogeneous: perforin-dependent antigen-specific memory CD8^+^ T cells promote Aβ plaque and Tau-like pathology and accumulate in sporadic AD brains, whereas GZMK^+^ and CXCR6^+^PD-1^+^ subsets appear to restrain pathological microglial accumulation and tau spread. Thus, distinct CD8^+^ T cell subsets exert divergent—even opposing—functions depending on subset, antigen specificity, and disease stage (104). Collectively, microglial dysfunction in AD is not determined exclusively by intracerebral protein pathology. Synergistic crosstalk between central glial responses, peripheral infection, vascular injury, and gut-derived signals jointly fuels disease progression. Accordingly, therapeutic strategies targeting microglia should consider both central and systemic immune-metabolic regulatory networks.

### 3.5 Cross-disease integration: shared and divergent microglial mechanisms in AD and MS

Although AD and MS are etiologically distinct—AD being an age-related proteinopathy and MS a chronic autoimmune demyelinating disorder of the CNS—microglia-driven neuroinflammation constitutes a convergent pathological axis in both diseases. Single-cell transcriptomic studies have shown that the DAM program first defined in AD models also emerges in experimental autoimmune encephalomyelitis (EAE) and in MS lesions, indicating that neuroinflammatory insults of very different origins funnel microglia into partially conserved reactive states (105–108). A systematic comparison of the two diseases therefore helps distinguish a druggable core of shared microglial mechanisms from disease-specific nodes that require tailored intervention.

Several core mechanisms delineated in the preceding sections of this review have well-documented counterparts in MS. First, the TREM2 axis is protective in both contexts: in AD, TREM2 drives the homeostatic-to-DAM transition and Aβ containment (Section 3.1), whereas in demyelinating disease TREM2 activation on microglia promotes myelin debris clearance and supports remyelination, and soluble TREM2 in cerebrospinal fluid has been explored as a biomarker in both disorders (109). Second, complement-mediated synaptic pruning, a central effector mechanism in early AD (Section 3.2), also operates in MS: C1q–C3 deposition marks synapses for microglial engulfment in the demyelinated hippocampus and cortex, and targeted complement inhibition at synapses prevents synapse loss in models of demyelinating disease (110, 111). Third, NLRP3 inflammasome signaling is activated in MS lesions and EAE, and pharmacological blockade of inflammasome effectors ameliorates disease severity, mirroring the NLRP3-centric amplification loop described in AD (112). Fourth, the lipid-laden, phagocytosis-impaired microglial state (LDAM: Sections 2.2 and 2.3) parallels the foamy, myelin-debris-laden microglia/macrophages that accumulate at the edge of chronic active MS lesions and lose degradative capacity with aging (113, 114). Fifth, iron-loaded microglia and ferroptotic stress, which surround Aβ plaques in AD (Section 3.3), are likewise a hallmark of MS, where iron-rimmed, slowly expanding lesions delineate compartments of sustained microglial injury (115, 116). Finally, interferon-responsive microglia (IRM; Section 2.3) and periphery-to-brain signaling through systemic inflammation and the gut–brain axis (Section 3.4) are prominent in both diseases.

Equally instructive are the divergences. MS is initiated by a breach of immune tolerance, with autoreactive CD4^+^ T cells and B cells orchestrating demyelination—explaining the efficacy of peripherally directed immunotherapies such as anti-CD20 antibodies—whereas in AD, adaptive immune infiltrates (including the GZMK^+^ and CXCR6^+^PD-1^+^ CD8^+^ T-cell subsets discussed in Section 3.4) appear to modulate, rather than initiate, a proteinopathy-driven process. The type-I interferon axis illustrates a further, striking divergence: IFN-β is an established disease-modifying therapy in MS, yet chronic IFN-I signaling in microglia exacerbates neuroinflammation and neurodegeneration in AD (Sections 2.3 and 3.4), demonstrating that the same microglial pathway can be therapeutic in one disease and deleterious in another. Disease stage and host age further distinguish the two conditions: remyelination failure and acute lesion activity dominate the young-adult context of MS, whereas SAM and immunometabolic decline shape the decades-long course of AD. These divergences also extend to therapeutic translation: MS already possesses an armamentarium of approved immunomodulatory drugs, several of which engage microglial biology—for example, Bruton’s tyrosine kinase inhibitors that act on both B cells and CNS-resident myeloid cells—whereas most microglia-targeted AD strategies remain preclinical (Section 4). This asymmetry identifies cross-disease repurposing—of NLRP3 inhibitors, complement modulators, and BTK inhibitors (117)—as a concrete translational opportunity.

Taken together, microglia function as a convergent execution hub of neuroinflammation across neurological diseases: AD and MS share a conserved mechanistic core—TREM2-dependent activation, complement-mediated synaptic pruning, NLRP3 signaling, lipid and iron dysregulation, and interferon-responsive states—while differing fundamentally in initiating drivers, adaptive-immune architecture, and therapeutic leverage points (**Table 1**). Recognizing this shared core situates the AD-centric synthesis of the present review within the broader neuroimmunology landscape and supports bidirectional knowledge transfer between the two fields: microglial mechanisms characterized in MS lesions can inform stage-specific targeting in AD, and vice versa.

## 4 Drug intervention: Microglia-targeted AD treatment strategies

Three complementary therapeutic axes currently guide microglia-directed AD drug development (**Figure 4**; **Table 3**). First, immunotherapy—via anti-Aβ antibodies or receptor modulation—boosts Aβ phagocytosis and clearance (4.1). Second, pathway blockade, targeting the NLRP3 inflammasome and complement, curbs hyperactive neuroinflammation (4.2). Third, metabolic reprogramming restores surveillance and clearance by remodeling glycolysis, the TCA cycle, and mitochondrial OXPHOS (4.3). These approaches target distinct functional layers—external recognition, internal signaling, and basal metabolism—allowing standalone use or synergistic combination. **Table 3** details the targets, mechanisms, and representative findings for each strategy in AD models. To render the strength of the underlying evidence explicit, we indicate throughout this section—and in the “Model” column of **Table 3**—whether each finding derives from human clinical studies, animal models, or *in vitro* experiments, and we distinguish clinically established treatments (agents approved for AD) from experimental approaches that remain at the preclinical or early clinical stage.

**Figure 4:**
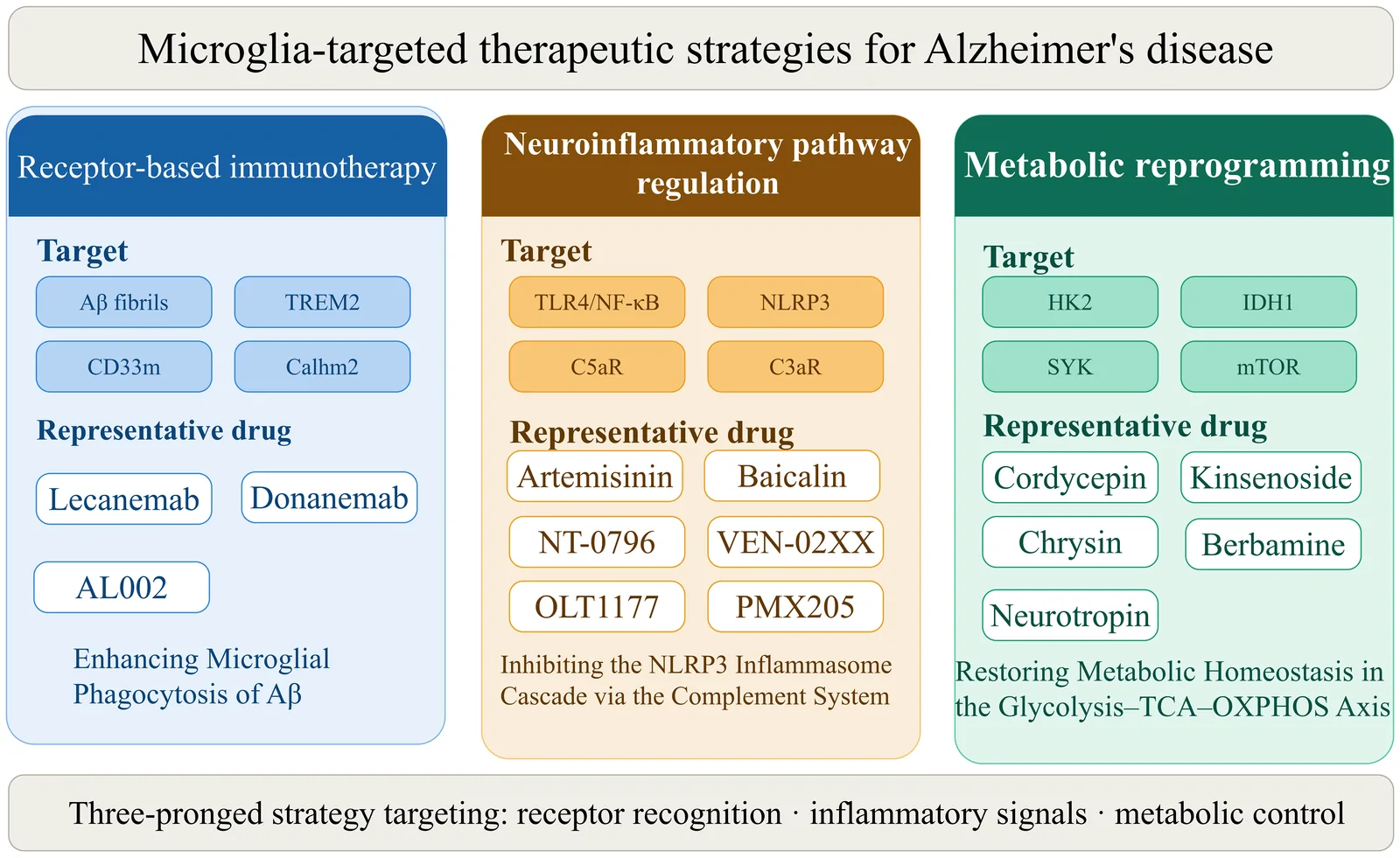
Classification of microglia-targeted therapeutic strategies for AD.

**Table 3:** Summary of microglia-targeted intervention strategies for AD.

Intervention	Target	Model	Key Findings	Reference
Lecanemab	soluble Aβ protofibrils/Fc receptor	Early-stage AD	Significantly improved CDR-SB scores at 18 months; ARIA-E occurred in 13.6% of patients.	(121)
Donanemab	N-truncated Aβ/Fc receptor	Early-stage AD	Significantly delayed clinical decline and reduced amyloid-β levels at 76 weeks.	(123)
AL002	TREM2	Early-stage AD	Achieved target engagement in the Phase II INVOKE-2 trial but did not meet the primary clinical endpoint.	(124, 125)
Artemisinin	TLR4/NF-κB	Aβ-treated C57 mice/BV2 microglia	Attenuated microglial oxidative stress and reversed spatial learning and memory deficits.	(130)
Oxysophoridine	TLR4/NF-κB	Aβ-induced BV2 cells	Suppressed oxidative stress and inflammatory responses.	(131)
Baicalin	NLRP3/TLR4/NF-κB	APP/PS1 mice; LPS/Aβ-stimulated BV2 cells	Reduced activated microglia and pro-inflammatory cytokines; alleviated spatial memory impairment.	(132)
Platycodin D	TLR4/NF-κB; Nrf2/HO-1	Aβ-induced BV2 cells	Reduced inflammatory factors and attenuated oxidative stress injury.	(133)
NT-0796	NLRP3	iPSC models/Non-human primates	A brain-penetrant prodrug activated by CES1, targeting microglia.	(134)
VEN-02XX	NLRP3	5×FAD/Rubicon KO mice	Post-symptom administration rescued cognition, reduced tau pathology, and prevented neuronal loss.	(135)
OLT1177 (Dapansutrile)	NLRP3	APP/PS1 mice	Oral NLRP3-specific inhibitor that improved spatial learning and memory.	(136)
PMX205	C5aR	AD mouse models (Tg2576)	Blocked C5aR, polarized microglia toward a DAM-like phenotype, and reduced Aβ burden and synaptic loss.	(137, 138)
C5a peptide vaccine	C5a	AD mouse models	Induced an anti-C5a immune response and inhibited microglial activation.	(139)
EP67	C5aR (Agonist)	5×FAD mice	Enhanced microglial Aβ phagocytosis, improved cognition, and reduced synaptic loss.	(141)
SB290157	C3aR	LPS-induced model	Reduced hippocampal neuroinflammation and inhibited loss of synaptic proteins.	(142)
Cordycepin	HKII/PDK2	AD cellular models	Inhibited glycolysis and promoted mitochondrial oxidative metabolism, driving M2-like polarization.	(147)
Deer Antler Uridine	HSP90/HIF-1α	AD mouse models	Regulated glycolysis and improved cognitive function.	(148)
Thymidine & 2’-Deoxyuridine	Glycolysis pathway	Aβ_25-35_-induced brain injury	Modulated glycolysis to reduce microglial activation and oxidative stress.	(149)
Kinsenoside	IDH1	AD models	Restored TCA cycle flux and reestablished immuno-metabolic homeostasis.	(150)
Chrysin	SYK	AD models	Reprogrammed microglial metabolism and function, alleviating AD symptoms.	(151)
Modified Xiong-Gui Decoction	TREM2	Cognitive impairment models	TREM2-mediated energy metabolic reprogramming promoted phagocytic clearance of Aβ.	(152)
Rapamycin	mTOR/Purine metabolism	ME/CFS patients (Phase II Pilot)	Inhibited microglial inflammation and improved mitochondrial energy metabolism.	(154)
Berbamine	mTOR (FRB domain)	AD models	Bound to the mTOR FRB domain, restoring autophagic flux and regulating polarization.	(155)
Neurotropin	FUS/Calhm2/EFhd2	AD models	Inhibited this axis to improve mitochondrial function and promote M1- to M2-like conversion.	(153)

### 4.1 Immunotherapy targeting Aβ and microglial receptors

Clinically established symptomatic AD pharmacotherapies fall into two approved classes: cholinesterase inhibitors (e.g., donepezil) and N-methyl-D-aspartate receptor antagonists (e.g., memantine); vitamin E is used only as an over-the-counter adjunct, not as an approved AD drug. Their clinical use rests on symptomatic efficacy demonstrated in human trials. Beyond this symptomatic benefit, preclinical studies have begun to characterize disease-modifying mechanisms: in cellular and animal models, donepezil suppresses microglial activation and neuroinflammation via MAPK/NLRP3/STAT3 signaling (118); memantine improves cognition in olfactory bulbectomized mice by inhibiting KATP channels (119); and vitamin E reduces lipid peroxidation and attenuates age-related decline (120). However, these mechanistic findings have not translated into clinically demonstrated disease modification; these agents remain symptomatic treatments, leaving a substantial unmet clinical need. Among the agents discussed in this review, only these symptomatic drug classes and the anti-Aβ monoclonal antibodies described below have established clinical efficacy in AD patients; all other strategies discussed in Sections 4.2 and 4.3 remain at the experimental or preclinical stage.

Anti-Aβ monoclonal antibodies (mAbs) have shifted the therapeutic paradigm from symptomatic management toward disease modification. Their putative mechanism—Fc receptor-mediated phagocytic clearance of Aβ by microglia—was established in animal models and *in vitro* phagocytosis assays before being validated in humans. In preclinical studies, chronic early intervention with anti-amyloid immunotherapy reduces plaque burden in a dose-dependent manner in transgenic mouse models of AD. In humans, lecanemab, a humanized mAb targeting soluble Aβ protofibrils, demonstrated significant benefits in the Phase III Clarity AD trial (n=1,795), improving Clinical Dementia Rating–Sum of Boxes (CDR-SB) scores after 18 months of treatment (121). It received accelerated FDA approval in January 2023 and was converted to traditional approval in July of the same year (122). Donanemab, which targets N-terminal pyroglutamate Aβ, significantly reduced amyloid levels, improved composite AD rating scale scores, and slowed clinical decline (assessed by CDR-SB) versus placebo over 76 weeks in the Phase III TRAILBLAZER-ALZ 2 trial (n=1,736) (123). Beyond direct Aβ clearance, *in vitro* studies show that the short CD33m splice variant—lacking the sialic acid-binding domain—stimulates microglial proliferation, migration, and phagocytosis of debris in cultured microglia, suggesting a potential protective effect that awaits validation in animal models and humans (21). Despite these cognitive benefits, safety concerns remain from the human trial data: in the pooled Clarity AD core study and open-label extension, amyloid-related imaging abnormalities with edema/effusion (ARIA-E) occurred in 13.6% of lecanemab-treated patients (16.8% in APOE4 carriers), while ARIA-H occurred in 18.5% (microhemorrhages 16.0%). Most findings were mild to moderate, with ARIA-E peaking between 3–6 months post-treatment (121). Thus, although anti-Aβ mAbs effectively reduce plaque load and slow cognitive decline, their net clinical benefit is constrained by microglial clearance efficiency and ARIA-related adverse events. Future strategies must leverage biomarkers for precise patient stratification and incorporate anti-inflammatory or vascular-protective adjunct therapies to broaden the therapeutic window and maximize clinical utility.

Attention has therefore increasingly turned to key regulatory receptors on microglia themselves. TREM2, a microglia-specific receptor, is a well-established AD risk gene in human genetic studies, and mechanistic work in mouse models has assigned it a central role in maintaining microglial homeostasis and mediating the transition to the DAM state (56). Therapeutic strategies targeting TREM2 aim to enhance microglial Aβ phagocytosis and neuroprotection; however, clinical translation has proven challenging. AL002, a TREM2-agonistic humanized IgG1 monoclonal antibody, demonstrated favorable central target engagement and safety in animal models and in a first-in-human Phase I study (124). Yet the Phase II INVOKE-2 trial (NCT04592874, n=381), reported in late 2024, showed that despite central target binding and pharmacodynamic responses, AL002 failed to significantly delay disease progression in early AD patients at the primary endpoint (CDR-SB), with no improvement in amyloid PET or cerebrospinal fluid biomarkers (125). Consequently, Alector terminated the long-term extension study. In a subsequent commentary, Colonna and Holtzman cautioned that this failure does not refute TREM2’s therapeutic potential but instead reflects suboptimal therapeutic design, not a failure of the target itself (126). Several factors may explain this outcome. First, the intervention window may have been suboptimal: TREM2 signaling drives the early transition of microglia toward the DAM state in a stage-dependent manner, so agonism initiated after symptoms are established may miss the period in which microglial engagement is most protective (56, 126). Second, target engagement does not guarantee efficacy: antibody-mediated agonism may not faithfully reproduce physiological TREM2 signaling—sustained receptor cross-linking could elicit downstream outputs distinct from endogenous ligand engagement—and the optimal dose and CNS exposure required for sustained receptor activation remain undefined (124–126). Third, INVOKE-2 enrolled an unselected early-AD population without stratification by TREM2 genotype or baseline microglial state, potentially diluting benefit in the subgroups most likely to respond (125, 126). Finally, the supportive preclinical evidence was generated predominantly in amyloid-driven mouse models, in which the human disease-associated microglial program—including TREM2-dependent responses—is only partially conserved, according to cross-species transcriptomic analyses (34, 35). These considerations argue that future TREM2-directed trials should prioritize earlier intervention, optimized dosing, biomarker-guided patient stratification, and rational combination regimens rather than single-agent agonism (126). Beyond TREM2, several other microglia-associated molecules have emerged as candidate targets, although—unlike the anti-Aβ antibodies discussed above—supporting evidence for these remains preclinical. In AD mouse models, Calhm2 is significantly upregulated, and both conventional and microglia-specific Calhm2 knockout reduces Aβ deposition, neuroinflammation, and cognitive impairment, identifying it as a potential therapeutic target (127). Findings from TSPO-deficient mice likewise indicate that this mitochondrial transporter regulates microglial phagocytosis and neuroinflammation, suggesting neuroprotective potential (128). *In vitro* and *in vivo* studies further show that pharmacological or genetic inhibition of USP25, a deubiquitinase within the ubiquitin–proteasome system, ameliorates amyloid pathology by regulating APP processing and Aβ generation (129). Collectively, microglia-directed AD interventions are evolving from single-pathway Aβ clearance toward multi-target, multi-pathway integrated regulation; however, it should be emphasized that, at present, Calhm2, TSPO, and USP25 are supported exclusively by animal and *in vitro* evidence and have not yet been validated in human studies. Future success will hinge on precisely defining the spatiotemporal windows of target engagement and rationally designing combinatorial therapeutic regimens.

### 4.2 Regulatory strategies targeting neuroinflammatory pathways

Neuroinflammation represents a central driver of microglia-mediated AD pathology. Targeting its key nodes—notably the NLRP3 inflammasome and complement system—offers a direct therapeutic avenue. Evidence from cultured microglia indicates that NLRP3 hyperactivation depends on TLR4/NF-κB signaling to upregulate NLRP3 and pro-IL-1β, with Aβ serving as a trigger for inflammasome assembly (50, 130).

Artemisinin attenuates Aβ-induced neuroinflammation and memory impairment in AD mouse models, whereas oxysophoridine suppresses Aβ-induced oxidative stress and inflammatory responses in BV2 microglia *in vitro*, both mechanistically linked to blockade of this pathway (130, 131). Baicalin mitigates microglia-mediated neuroinflammation and cognitive impairment in AD mouse models, whereas platycodin D reduces pro-inflammatory cytokine production and oxidative injury in BV2 cells *in vitro* (132, 133). The direct NLRP3 inflammasome-targeting agent NT-0796, an ester-containing prodrug NLRP3 inhibitor with efficient blood–brain barrier penetration—confirmed in iPSC-derived human brain endothelial models and non-human primate pharmacokinetic studies—is metabolized within the brain parenchyma by carboxylesterase-1 (CES1), which is predominantly expressed in microglia, to its active form, NDT-19795 (134). In the 5×FAD/Rubicon KO AD mouse model, VEN-02XX, a novel selective brain-penetrant small-molecule NLRP3 inhibitor, demonstrated efficacy, rescuing cognitive deficits, reducing neuronal loss, and curtailing reactive microgliosis, neuroinflammation, and tau pathology even when administered after symptom onset (135). OLT1177 (Dapansutrile), an oral NLRP3-specific inhibitor, improves spatial learning and memory in the APP/PS1 model (136). Although these findings provide encouraging proof-of-concept validation, the supporting evidence derives entirely from *in vitro* systems and animal models, and no NLRP3 inhibitor has yet demonstrated clinical efficacy in AD. Rigorous evaluation of their efficacy and safety in AD patients remains an essential prerequisite for clinical translation.

The complement system exerts dual functions in microglia-mediated synaptic pruning and neuroinflammation. In AD mouse models, the C5a receptor antagonist PMX205 significantly suppresses neuroinflammation, reduces multiple pathological markers, and improves behavioral deficits by blocking C5aR1, highlighting the neuroprotective potential of complement pathway targeting (137). Subsequent studies in Tg2576 mice confirmed that PMX205 not only reduced dystrophic neurons and amyloid burden but also alleviated excessive presynaptic loss associated with cognitive impairment; concurrently, microglial gene expression shifted toward a DAM-like phenotype, closely paralleling the observed neuroprotective effects (138). Additionally, a C5a peptide vaccine induces a specific immune response against the pro-inflammatory molecule C5a, inhibiting microglial activation and reducing neuroinflammation in AD mouse models, thereby ameliorating neurological dysfunction and AD-related symptoms (139). A recent review underscores the protective role of C5aR1 antagonists—exemplified by PMX205—in reducing neuroinflammation and preserving synaptic function, highlighting the C5–C5aR1 axis as a therapeutic target for neurodegenerative diseases while emphasizing the need for novel C5aR1 antagonist development (140). In contrast, the modified C5a receptor agonist EP67 improves cognitive function and reduces synaptic loss in the 5×FAD model by enhancing microglial phagocytosis of both fibrillar and non-fibrillar Aβ (141). The C3a receptor antagonist SB290157 (trifluoroacetic acid salt) attenuates LPS-induced hippocampal neuroinflammation and prevents synaptic protein loss via the complement–microglia axis, thereby preserving cognition (142). Collectively, this preclinical evidence—derived exclusively from animal models and *in vitro* experiments—supports the therapeutic potential of targeting the NLRP3 inflammasome and complement system to modulate neuroinflammation in AD; to date, no complement-modulating agent has yet been evaluated in AD patients, let alone demonstrated clinical efficacy. However, clinical translation remains hindered by challenges including target dual functionality, off-target effects, and safety considerations that demand rigorous evaluation.

### 4.3 Strategies targeting microglial metabolic remodeling

Metabolic reprogramming of microglia has emerged as a new frontier in AD research. Unlike strategies directly targeting Aβ receptors or suppressing inflammatory pathways, metabolic remodeling aims to restore microglial energy homeostasis and physiological function, breaking the vicious cycle of chronic neuroinflammation at its source. It should be noted at the outset that the strategies discussed in this section are supported almost entirely by preclinical evidence—derived from AD mouse models and *in vitro* systems—and none has yet demonstrated therapeutic efficacy in AD patients. In AD, microglial mitophagy is significantly impaired, with disruption of the PINK1–Parkin pathway leading to ROS accumulation and activation of the innate immune cascade (143, 144). Concurrently, microglia exhibit glycolytic reprogramming, characterized by a compensatory increase in glycolytic flux and a parallel decline in OXPHOS—a metabolic shift directly demonstrated in human postmortem brain tissue: multiplex immunohistochemistry of hippocampal–entorhinal cortex from AD patients revealed PKM2^+^ glycolytic microglia accumulating around Aβ plaques, p-Tau tangles, and cerebral vasculature, exhibiting phagocytic exhaustion and impaired chemotaxis (145). Hexokinase 2 (HK2), a rate-limiting glycolytic enzyme, plays a pivotal role in this process; HK2 is upregulated in plaque-associated microglia in both 5×FAD mice and postmortem human AD brains, and, in mouse studies, its dosage critically influences microglial activation states and AD progression, suggesting that glycolytic nodes—rather than terminal inflammatory effectors—may represent more effective intervention targets (146). Based on this rationale, multiple natural compounds have demonstrated neuroprotective effects by targeting key glycolytic regulators. Cordycepin simultaneously targets HK2 and PDK2, curbing excessive glycolysis while promoting mitochondrial oxidative metabolism and driving microglial polarization toward an anti-inflammatory phenotype in APP/PS1 mice and cultured microglia (147). Deer antler uridine regulates glycolysis via the HSP90/HIF-1α axis, improving cognitive function in AD mice (148). Thymidine and 2′-deoxyuridine alleviate microglial activation and oxidative injury by modulating glycolytic pathways in an Aβ25-35-induced brain injury model (149). At the TCA cycle level, IDH1—a key rate-limiting enzyme of this cycle—is upregulated in microglia from both AD patients and 5×FAD mice; kinsenoside, a natural IDH1 inhibitor identified through structure-based screening, restores tricarboxylic acid flux by targeting IDH1, reconstructing microglial immune–metabolic homeostasis and improving cognition in 5×FAD mice (150). Chrysin reprograms microglial metabolism and function via SYK kinase inhibition, alleviating AD symptoms in AD mice, with mechanistic confirmation in BV2 microglia *in vitro* (151). Collectively, these strategies share a common paradigm: rather than directly suppressing terminal inflammatory signals, they restore microglial physiological surveillance and clearance by repairing the integrity of core metabolic pathways—a paradigm that holds promise for addressing AD pathogenesis upstream of irreversible neuroinflammatory damage.

Metabolic remodeling is closely coupled with phagocytic function. Xiong-gui Decoction promotes microglial Aβ clearance and alleviates cognitive impairment via TREM2-mediated energy metabolism reprogramming in AD mouse models, suggesting that the receptor–metabolic axis may serve as a critical node integrating immune surveillance and energy regulation (152). Neurotropin improves mitochondrial function via the FUS/Calhm2/EFhd2 axis and promotes microglial polarization from a pro-inflammatory to an anti-inflammatory phenotype (153). Collectively, these strategies, despite differing in targets and administration, share a common therapeutic philosophy: rather than directly extinguishing inflammation, they restore microglial intrinsic metabolism and clearance capacity—offering a paradigm shift toward disease modification in AD. However, current evidence in AD remains confined to *in vitro* studies and animal models. The blood–brain barrier permeability and target selectivity of natural compounds require further elucidation. Moreover, metabolic reprogramming exhibits cell-type specificity; off-target effects on neurons and astrocytes must be carefully considered when designing microglia-directed interventions. Future research should prioritize the development of microglia-specific metabolic modulators and multi-target combination regimens to accelerate the translation of metabolic remodeling from proof-of-concept to clinical application.

## 5 Non-pharmacological interventions: from lifestyle modulation to microglial remodeling

Given the limited clinical efficacy of pharmacological treatments for AD over recent decades, non-pharmacological strategies have garnered increasing scholarly attention. Approaches such as physical exercise (156), music therapy (157), and transcranial magnetic stimulation combined with cognitive training (158) have demonstrated positive effects on AD-related symptoms. Unlike drug-based therapies, these interventions modulate modifiable lifestyle factors and environmental exposures systemically with minimal side effects, offering a vital complementary strategy to slow disease progression. As the CNS’s primary innate immune cells, microglia act as key sensors linking peripheral environments to brain immune homeostasis. Environmental pollution and modern lifestyle factors—including chronic stress, poor diet, and sleep/circadian rhythm disturbances—can drive neuroinflammation by altering microglial function and phenotype, ultimately contributing to cognitive impairment (159). This section examines these pathways across four dimensions: exercise, diet and intestinal microecological regulation, circadian rhythm and sleep, and physical factor interventions (**Figure 5**; **Table 4**).

**Figure 5:**
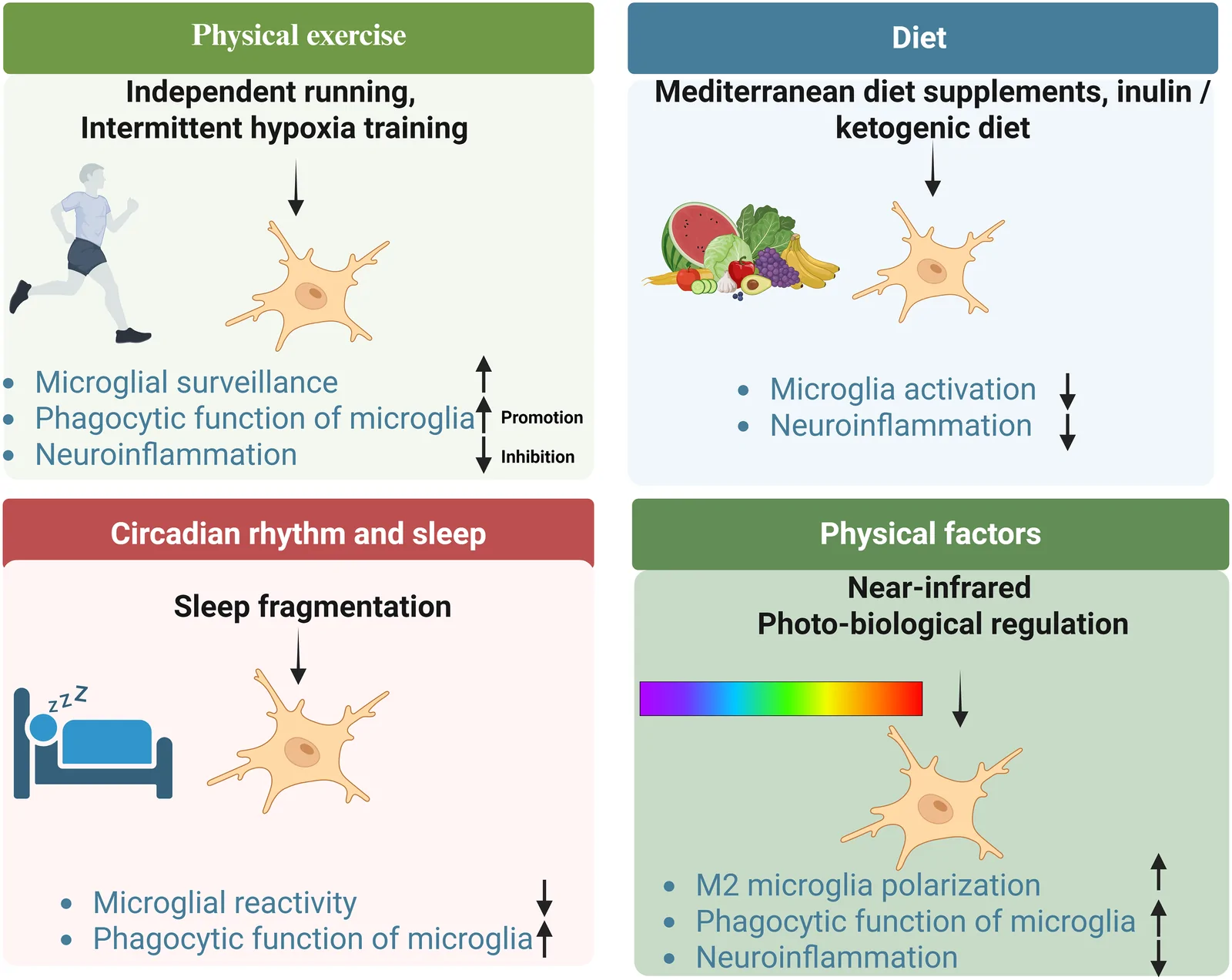
Effects of non-pharmacological intervention strategies on microglial phenotype and function. Created with BioRender.com.

**Table 4:** Effects of non-pharmacological interventions in AD.

Intervention	Target/Mechanism	Model	Key Findings	Reference
Long-term treadmill running	TREM2/SYK	APP/PS1 mice	Enhanced microglial glucose metabolism and morphological plasticity; improved Aβ phagocytosis and cognitive function.	(160)
Long-term voluntary wheel running	AGE/RAGE–complement pathway	APP/PS1 mice	Reduced CD68/Iba-1 expression, inhibited the AGE/RAGE and C1q/C3 cascades, and protected hippocampal dendritic spines.	(161)
Voluntary wheel running	Oligodendrocyte–myelination axis	APP/PS1 mice	Reduced plaque-associated microglial activation and promoted oligodendrocyte maturation and myelination in the mPFC.	(162)
Intermittent hypoxia therapy	TFEB/Autophagy–lysosomal pathway	APP/PS1 mice	Upregulated TFEB to enhance DAM-mediated Aβ degradation and alleviate cognitive impairment.	(163)
Intermittent hypoxia training	VPS35/TREM2	APP/PS1 mice	Upregulated VPS35 to enhance TREM2 intracellular recycling and Aβ endocytosis; the protective effect was abolished by VPS35 knockout.	(164)
Ketogenic diet	Gut microbiota	5×FAD mice	Reduced microglial activation, improved cognition, and decreased Aβ deposition and neuroinflammation.	(165)
Spray-dried porcine plasma supplementation	Microbiome–gut–brain axis (MGBA)	SAMP8 mice	Increased gut probiotic abundance and attenuated microglial activation and neuroinflammation.	(166, 167)
Mediterranean diet supplement (Neurosyn240)	MGBA/Serotonin/Bile acids	5×FAD mice	Remodeled gut microbiota, elevated serotonin, reduced bile acids, and decreased Iba-1^+^ microglial activation and Aβ deposition.	(169)
Dietary inulin supplementation	Akkermansia/MGBA	5×FAD mice	Increased gut Akkermansia abundance, alleviated microgliosis, and reduced Aβ plaque burden.	(170)
Sleep fragmentation	Microglial senescence	Elderly humans (MAP/ROS cohort)	Accelerated microglial senescence and morphological activation, increasing the risk of cognitive decline and dementia.	(173)
REV-ERB inhibition (SR8278) or Rev-erbα knockout	REV-ERBs	5×FAD mice	Promoted M2-like polarization, enhanced Aβ phagocytosis, and reduced amyloid plaque deposition.	(174)
Near-infrared light	Mitochondrial oxidative metabolism	Aβ-treated microglia	Suppressed ROS production, shifted metabolism from glycolysis toward mitochondrial oxidation, and enhanced neuronal survival.	(175)
Far-infrared irradiation	ATP production	APP/PS1 mice; primary microglia	Induced microglial phagocytic clearance of Aβ, reduced Aβ burden, and improved cognition.	(176)
Low-dose ionizing radiation	TREM2	5×FAD mice; LPS-stimulated BV2 cells	Promoted M2-like polarization via TREM2 upregulation, inhibited Aβ deposition, and ameliorated cognitive deficits.	(177)
Photobiomodulation (PBM, 808 nm)	OXPHOS/HK2–glycolysis axis	APP/PS1 mice	Promoted M2-like polarization, enhanced Aβ phagocytosis, improved cognition, and reduced neuronal apoptosis.	(178)

### 5.1 Exercise intervention

Exercise constitutes one of the most robustly evidenced non-pharmacological strategies for ameliorating cognitive impairment in AD. In APP/PS1 mice, long-term voluntary running promotes glucose metabolism and morphological plasticity in hippocampal microglia while enhancing Aβ phagocytosis via the TREM2/SYK signaling pathway. This effect—attributable primarily to increased microglial survival rather than proliferation—ultimately improves cognitive function (160). Mechanistic insights have since expanded. Wang et al. reported that four months of voluntary running in 10-month-old APP/PS1 mice significantly suppressed hippocampal AGE/RAGE signaling and the C1q/C3 complement system, alongside reduced expression of microglial activation markers (CD68, Iba-1) and mitigated dendritic spine loss. These findings indicate that exercise preserves hippocampal synaptic integrity via the AGE/RAGE–complement axis (161). Extending these observations, Pan et al. demonstrated that three months of voluntary running not only curbed Aβ plaque deposition in the medial prefrontal cortex but also enhanced oligodendrocyte maturation and myelination surrounding these plaques. Thus, exercise-derived neuroprotection transcends the hippocampus, engaging broader brain regions and diverse glial populations (162). Intermittent hypoxic training (IHT) offers an alternative exercise-adaptation paradigm with distinct microglial implications. Wang et al. initially showed that IHT potentiates Aβ degradation in plaque-associated microglia via TFEB-mediated activation of the autophagy–lysosomal pathway, alleviating cognitive deficits in APP/PS1 mice (163). Extending this, the same group found that IHT upregulates VPS35 through TFEB-driven transcription, enhancing TREM2 intracellular recycling and membrane expression to promote Aβ endocytosis by DAM. Notably, these protective effects were abolished in microglia-specific VPS35 knockout mice, confirming the VPS35–TREM2 axis as the core effector mechanism of IHT (164). Collectively, these findings establish that exercise remodels microglial phagocytic and metabolic functions via converging yet distinct molecular cascades—the TREM2/SYK, AGE/RAGE–complement, and TFEB–VPS35–TREM2 axes. Importantly, continuous voluntary running and IHT appear to engage different functional nodes within these networks, providing a molecular framework to guide the individualized design of exercise-based therapeutic regimens for AD.

### 5.2 Diet and intestinal microecological regulation

As a modifiable lifestyle factor, diet profoundly influences microglial function via the microbiome–gut–brain axis (MGBA). Ketogenic diets reduce neuroinflammation and improve cognition by modulating gut flora, curtailing amyloid plaque deposition and microglial activation (165). Dietary supplementation with spray-dried porcine plasma boosts intestinal probiotic abundance, attenuating microglial activation and neuroinflammation through MGBA (166, 167), while DASH and Mediterranean dietary patterns confer established prophylactic benefits against AD (168). Recent mechanistic studies have elucidated these pathways at the molecular level. Connell et al. developed Neurosyn240, a supplement rich in key bioactive components of the Mediterranean diet. In 5×FAD mice, it markedly reshaped gut flora composition, elevated circulating serotonin, and reduced bile acid concentrations (e.g., lithocholic acid), consequently lowering hippocampal Aβ deposition and Iba-1^+^ microglial activation. Hippocampal RNA sequencing further revealed upregulation of Aβ-clearance genes (e.g., *Lrp2*, *Clu*), underscoring the microbiota–metabolite–brain axis as a critical bridge in microglial regulation (169). Using single-cell RNA sequencing, Wang et al. performed whole-brain transcriptomic analysis at single-cell resolution in 5×FAD mice. They found that dietary inulin specifically enriched intestinal *Akkermansia muciniphila*, reduced forebrain Aβ plaque load, and mitigated microglial proliferation, although spatial learning and memory remained unaffected (170). This study provides a valuable single-cell atlas for deciphering the differential effects of high-fiber diets across various brain cell types.

Conversely, poor dietary habits exacerbate AD pathology via microglia-mediated mechanisms. In the *App^NL-G-F^* knock-in mouse model, Yang et al. demonstrated that a long-term high-fat diet (HFD) accelerated Aβ deposition, weakened microglial recruitment and phagocytosis toward plaques, and induced massive intracellular lipid droplet accumulation. Lipidomics revealed a synergistic increase in cortical cholesteryl esters driven by both HFD and AD pathology. *In vitro*, oleic acid—a primary HFD component—downregulated cholesterol efflux and phagocytosis-related genes in MG6 microglia, identifying HFD-induced disruption of brain cholesterol homeostasis as a key mechanism that impairs microglial function and hastens AD progression (171). Collectively, these findings illustrate a bidirectional regulatory capacity: healthy dietary patterns foster a protective microglial phenotype through MGBA, whereas poor nutrition triggers dysfunction via lipid metabolic disturbances. Nevertheless, translating these preclinical insights into effective nutritional intervention strategies must account for individual metabolic variability, baseline gut microbiota composition, and optimal intervention timing.

### 5.3 Circadian rhythm and sleep regulation

Disruption of sleep–wake rhythms constitutes a key modifiable risk factor for AD. In 3×Tg-AD mice, four weeks of chronic sleep fragmentation increased microglial activation and pro-inflammatory cytokine levels, thereby stimulating hippocampal neuroinflammation and exacerbating Aβ accumulation (172). Parallel clinical findings confirm that sleep fragmentation correlates with cognitive decline and increased dementia risk in the elderly, an association potentially mediated by microglial senescence (173). At the molecular level, pharmacological inhibition or genetic knockout of REV-ERBs accelerates microglial clearance of Aβ and reduces amyloid plaque burden (174). This indicates that circadian clock signals not only govern microglial phagocytosis but also directly influence the dynamics of Aβ metabolism. Despite these promising mechanistic insights, specific interventions directly targeting microglial circadian machinery remain undeveloped. Moreover, critical parameters—including the optimal time window, intervention duration, and potential combination strategies with existing pharmacotherapies—warrant systematic investigation in future translational studies.

### 5.4 Physical factor intervention

Various physical modalities exert neuroprotective effects by modulating microglial metabolism and functional status. Near-infrared irradiation curbs reactive oxygen species production in Aβ-stimulated microglia, shifting their metabolic profile from glycolysis toward mitochondrial oxidative metabolism, thereby reducing Aβ-induced cytotoxicity and enhancing neuronal survival (175). Far-infrared irradiation augments ATP production to promote microglial phagocytosis and reduce cerebral Aβ burden (176), while low-dose ionizing radiation upregulates TREM2 expression to drive M2-like polarization, inhibit Aβ deposition, and ameliorate cognitive impairment (177).

Photobiomodulation (PBM), a non-invasive physical therapy, has demonstrated significant potential in AD models. Six weeks of continuous 808 nm PBM in APP/PS1 mice enhanced Aβ phagocytosis and clearance by modulating mitochondrial energy metabolism—promoting OXPHOS and inhibiting glycolysis—thereby polarizing microglia toward an anti-inflammatory phenotype. Notably, pharmacological inhibition of HK2 with 3-bromopyruvate partially mimicked these effects, identifying the HK2–glycolysis axis as a critical node in PBM-driven metabolic reprogramming (178). A systematic review further highlights PBM’s multifaceted protective actions—enhancing mitochondrial function, suppressing neuroinflammation, and improving cerebral perfusion and lymphatic drainage—with both transcranial and systemic PBM holding translational promise (179). Mechanistically, this PBM-induced metabolic shift parallels the drug-based strategy discussed in Section 4.3, as both converge on the glycolysis–OXPHOS switch. However, PBM achieves this metabolic correction through physical rather than pharmacological means, providing a theoretical rationale for combinatorial intervention strategies. Nonetheless, key therapeutic parameters—including wavelength, power density, and irradiation duration—remain unstandardized. Moreover, the current evidence rests predominantly on animal models; rigorous evaluation of efficacy and safety in human subjects is imperative before clinical translation.

## 6 Summary and prospect

This review provides a systematic synthesis of the multidimensional functions of microglia in AD and the corresponding intervention strategies. The field has shifted from the classical M1/M2-like polarization dichotomy toward a functional heterogeneity paradigm, driven largely by single-cell omics. The identification of distinct subpopulations—including DAM, MGnD, LDAM, and SAM—has deepened our understanding of microglial state trajectories and their stage-dependent functional transitions. At the mechanistic level, a complex molecular network orchestrates microglial contributions to AD progression. This network centers on the Aβ/Tau–TREM2–APOE signaling hub, the complement–NLRP3 inflammatory amplification loop, immunometabolic reprogramming, and ferroptosis. Meanwhile, peripheral inflammation, cerebrovascular injury, and the gut–brain axis act as dynamic microenvironmental modulators. These multilayered interactions not only underscore the central role of microglia but also imply that single-target strategies are unlikely to cover the full pathogenic spectrum. On the translational front, anti-Aβ monoclonal antibodies (e.g., lecanemab and donanemab) have ushered AD treatment into the disease-modifying era. However, safety concerns such as ARIA and the limited efficiency of spatial clearance indicate that Aβ removal alone cannot halt disease progression. The phase II failure of the TREM2 agonist AL002 further highlights the need to optimize therapeutic timing, dosing regimens, and combinatorial approaches for receptor-targeted strategies. Metabolic remodeling offers a complementary avenue by restoring microglial energy homeostasis, while non-pharmacological interventions—including exercise, dietary modulation, circadian regulation, and photobiomodulation—provide systemic, low-side-effect adjuncts that may slow AD progression.

Despite these advances, translating this progress into clinical practice must be tempered by the limitations of the current evidence base. Much of our mechanistic understanding derives from transgenic mouse models (e.g., APP/PS1, 5×FAD, 3×Tg-AD) that overexpress mutant human APP or presenilins, producing artificially high and rapidly progressive Aβ pathology that does not reproduce the decades-long course, mixed proteinopathies, aging-related immunosenescence, systemic comorbidities, and vascular injury of sporadic human AD. Microglial biology also differs substantially between species—rodent disease-associated microglial signatures are only partially conserved in humans, and TREM2-dependent and TREM2-independent responses documented in human single-nucleus studies are not fully recapitulated in mouse models—while a considerable share of functional evidence still comes from BV2 cells or primary mouse microglia *in vitro*, which do not faithfully preserve aged, disease-associated human states. These species- and system-level constraints translate into at least five knowledge gaps that must be addressed before microglia-targeted therapies can reach clinical practice: (i) regional heterogeneity—microglial states differ markedly across hippocampal, cortical, white-matter, and perivascular niches, yet most interventions are evaluated without regional resolution; (ii) disease-stage dependence—the same microglial subset can be protective or injurious at different stages, and the spatiotemporal windows for intervention remain poorly defined, as starkly illustrated by the AL002 experience discussed above; (iii) target selectivity—most candidate drugs act on pathways that are also operative in astrocytes, neurons, and peripheral myeloid cells, and truly microglia-selective delivery strategies are still in their infancy; (iv) host context—APOE genotype and biological sex strongly modulate microglial responses but are rarely integrated into preclinical study design; and (v) biomarker-guided trials—the field still lacks validated markers and imaging tools to resolve microglial states in living patients, and without them for patient stratification and pharmacodynamic monitoring, clinical trials risk enrolling heterogeneous populations in which genuine efficacy is diluted. Closing these gaps will require a coordinated shift toward human single-nucleus and spatial transcriptomic datasets, humanized and knock-in models such as *App^NL-G-F^*, iPSC-derived microglia, and state-specific PET and fluid biomarkers—together enabling region-resolved disease modeling and biomarker-driven early-intervention trials that bridge the species- and stage-gap between mice and patients.

In conclusion, a comprehensive dissection of AD-associated microglial phenotypes and their regulatory networks will continue to uncover novel pathogenic mechanisms and therapeutic targets. Despite current challenges, continued exploration of integrated strategies—guided by human-validated targets, region- and stage-matched interventions, and biomarker-driven trial designs—holds promise for ultimately achieving safe and effective treatments for this devastating disease.
